# Carbon Electrode Sensor for the Measurement of Cortisol with Fast-Scan Cyclic Voltammetry

**DOI:** 10.3390/bios13060626

**Published:** 2023-06-06

**Authors:** Michelle Hadad, Nadine Hadad, Alexander G. Zestos

**Affiliations:** Department of Chemistry, American University, Washington, DC 20016, USA; mh4093a@american.edu (M.H.); nh3965a@american.edu (N.H.)

**Keywords:** cortisol, carbon fiber microelectrode, fast scan cyclic voltammetry

## Abstract

Cortisol is a vital steroid hormone that has been known as the “stress hormone”, which is elevated during times of high stress and anxiety and has a significant impact on neurochemistry and brain health. The improved detection of cortisol is critically important as it will help further our understanding of stress during several physiological states. Several methods exist to detect cortisol; however, they suffer from low biocompatibility and spatiotemporal resolution, and they are relatively slow. In this study, we developed an assay to measure cortisol with carbon fiber microelectrodes (CFMEs) and fast-scan cyclic voltammetry (FSCV). FSCV is typically utilized to measure small molecule neurotransmitters by producing a readout cyclic voltammogram (CV) for the specific detection of biomolecules on a fast, subsecond timescale with biocompatible CFMEs. It has seen enhanced utility in measuring peptides and other larger compounds. We developed a waveform that scanned from −0.5 to −1.2 V at 400 V/s to electro-reduce cortisol at the surface of CFMEs. The sensitivity of cortisol was found to be 0.87 ± 0.055 nA/μM (n = 5) and was found to be adsorption controlled on the surface of CFMEs and stable over several hours. Cortisol was co-detected with several other biomolecules such as dopamine, and the waveform was fouling resistant to repeated injections of cortisol on the surface of the CFMEs. Furthermore, we also measured exogenously applied cortisol into simulated urine to demonstrate biocompatibility and potential use *in vivo*. The specific and biocompatible detection of cortisol with high spatiotemporal resolution will help further elucidate its biological significance and further understand its physiological importance and impact on brain health.

## 1. Introduction

Cortisol is an essential steroid hormone, synthesized from cholesterol, more often known as the “stress hormone” due to its connection to the body’s response to anxiety. Cortisol is a known potential biomarker for psychological stress detection, and this psychological stress is caused by everyday lifestyle activities [1]. Being able to understand how cortisol functions in the body can help individuals balance their hormones and achieve optimal brain health. Cortisol is one of the many steroid hormones made in the adrenal glands [2]. Moreover, it is the main glucocorticoid released from the adrenal cortex, specifically, the zona fasciculata layer of the cortex [2]. The secretion of cortisol occurs with the control of the hypothalamus, pituitary gland, and adrenal gland (a combination of these glands referred to as the HPA axis). The main functions of cortisol in the human body include immune response, stress response, and glucose and protein homeostasis. Cortisol has great effects on blood sugar, metabolism, inflammation, and memory formulation [3]. High levels of cortisol may be linked with adverse brain health conditions such as anxiety and depression. Increased levels are associated with some tumors on the pituitary or adrenal glands that contribute to a sickness known as Cushing syndrome. Low levels of cortisol may potentially cause Addison’s disease or primary adrenal insufficiency [4]. Understanding cortisol’s function is essential when discussing further implications of neurochemistry on brain health.

Cortisol can be secreted in response to stress and many other vital body functions. Currently, many methods exist to measure cortisol in urine, saliva, or serum to serve as a diagnostic for Cushing’s syndrome when glucocorticoid levels are in excess. Biochemical assays also exist for plasma adrenocorticotropic hormone (ACTH) that is used for localization studies as well as the management of Cushing’s syndrome [5]. Scientists have also optimized the detection of cortisol in human sweat, which is another application of the measurement of cortisol [6]. Sweat and saliva samples were collected from subjects after intense exercise and analyzed using salivary enzyme-linked immunosorbent assay (ELISA), which measured hydrocortisone levels in human hair. Chromatographic techniques have also been traditionally used to detect cortisol [6]. On the other hand, the electrochemical immunosensing of cortisol is a relatively new advancement made toward point-of-care (POC) detection of cortisol. It is simple, low cost, and efficacious due to its high sensitivity and selectivity of detection, especially with respect to cortisol [1]. This electrochemical detection has also been used for measuring cortisol using anti-cortisol antibodies with immobilized nanostructures such as self-assembled monolayers and polymers for POC sensors. Other materials such as hydrogels offer other specific advances in sensing such as POC environmental [7], self-healing multifunctional sensing [8], human motion [9], antibiotics [10], and other applications [11]. These methods all provide advances in the measurement of cortisol but lack the specificity, selectivity, and rapid response time of other techniques such as voltammetric measurements.

For future clinical applications, POC sensors are highly specific and uncomplicated. An aptamer was modified to allow cortisol to competitively bind, therefore measuring levels in various biological samples such as saliva and serum [12]. Using square wave voltammetry (SWV), they applied a potential waveform scanning from −0.5 to −1.2 V toward the positive direction. Cortisol has also been detected using electrochemical methods such as using glass carbon electrodes (GCE) within a bonded microwell or nanoslit device [12]. Several electrochemical methods have been developed to detect and measure cortisol. Such methods include different assays to measure cortisol in specific samples such as either in sweat or hair. An automated electro-chemiluminescent immunoassay is a unique methodology that allows for a quicker and more precise response to a larger sample of cortisol [13]. This technique is advantageous to mass spectrometry, which is a common and more sensitive method of detection since it is less expensive and more accessible. The immunoassay was able to detect various levels of cortisol in human hair of both low and highly stressed individuals. Cortisol has also been measured from secretions found in bio-fluids such as sweat with modified conductive carbon fibers. A carbon electrode was functionalized with ellipsoidal Fe_2_O_3_ with 1-Ethyl-3-(3-dimethylaminopropyl) carbodiimide (EDC) and N-Hydroxysuccinimide (NHS) chemistry to immobilize the antibodies specific to cortisol [14]. Another study detected changing levels of cortisol by its binding to MoS_2_ nanosheets then measured with electrochemical impedance spectroscopy [15]. Furthermore, wireless aptamer field effect transistors (FETs) have also been used for the sensitive detection of cortisol [16]. These studies all demonstrate a variety of different methods to measure cortisol *ex vivo* and provide clinical implications on how cortisol measurements may be used to develop non-invasive POC devices for stressed individuals.

Recently, carbon fiber microelectrodes (CFMEs) have been utilized with fast-scan cyclic voltammetry (FSCV) for the measurements of several biologically active neurotransmitters [17,18]. By scanning to higher voltages and back at relatively fast scan rates greater than 100 V/s, the analytes are oxidized and then reduced at the electrode surface [19,20]. Using CFMEs and FSCV has several advantages over other techniques as they have fast electron transfer kinetics [21], high spatiotemporal resolution [22], biocompatibility [23] and are minimally invasive. CFMEs have also been used for the detection of other molecules in addition to neurotransmitters such as metals [24,25,26] and more complex molecules such as amino acids [27,28] and neuropeptides [29]. Through waveform modification, it has been shown that the detection of several molecules can be optimized with FSCV.

Here, we utilize FSCV, an electroanalytical technique that allows for the redox measurement of cortisol on carbon fiber microelectrodes (CFMEs). As opposed to other electroanalytical techniques such as amperometry, FSCV affords both the sensitive and selective detection of biomolecules based on the shape and the position of the cyclic voltammogram, which is a chemical fingerprint for detection. We applied a waveform on the CFMEs that scanned from −0.5 to −1.2 V at 400 V/s, which reduced cortisol on the surface of the CFMEs. Cortisol was found to be adsorption controlled onto the surface of CFMEs and stable at the surface of the electrode over several hours. Cortisol did not foul the surface of the microelectrode with the waveform applied and was co-measured with other biomolecules such as neurotransmitters, dopamine, and serotonin. Proof of concept measurements were performed in biological fluid to illustrate potential *in vivo* applicability. The biocompatible measurement of cortisol at a fast, subsecond timescale will potentially enhance the detection of cortisol and help further elucidate its physiological role *in vivo* on brain health and neurochemistry.

## 2. Materials and Methods

### 2.1. Chemicals

All of the chemicals such as cortisol were procured from Sigma-Aldrich, USA (Milwaukee, WI, USA) and were used to prepare stock solutions as received. Each 10 mM stock solution was prepared in 0.1 M perchloric acid and diluted with buffer (145 mM NaCl, 2.68 mM KCl, 1.4 mM CaCl_2_, 1.01 mM MgSO_4_, 1.55 mM Na_2_HPO_4_, and 0.45 mM NaH_2_PO_4_ with pH adjusted to 7.0). Epon 828 Epoxy was obtained from Miller–Stephenson, and diethylenetriamine hardener was obtained from Sigma-Aldrich. All aqueous solutions were made with deionized water (Millipore, Billerica, MA, USA). A 10 millimolar (mM) stock solution of cortisol was made in ethanol and then diluted in buffer. The cortisol stock solution was periodically diluted in buffer to several concentrations used throughout the study.

### 2.2. CFME Fabrication

Carbon fiber microelectrodes (CFME) were fabricated as previously described [30] by aspirating a carbon fiber into a 1.2 mm × 0.68 mm, glass capillary. The glass capillary was pulled to a fine taper with a Narishige PC-100 vertical capillary puller. The electrodes were trimmed to approximately 100 microns under the microscope to a protruding length. The electrodes were then epoxied with a 3:1 mixture of EPON 828 hardener and diethylenetriamine (DETA) and cured in the oven for approximately 3 h at 125 °C.

### 2.3. Fast-Scan Cyclic Voltammetry

Fast-scan cyclic voltammetry (FSCV) measurements were performed with a WaveNeuro FSCV system (Durham, NC, USA), and a 5 MΩ headstage from Pine Instruments. The runs were performed with high-definition cyclic voltammetry (HDCV) software, which records the data and then could be used for analysis. A carbon fiber electrode is used as the working electrode, where the reaction will occur, and a silver–silver chloride (Ag/AgCl) wire is used as the reference electrode, which provides constant concentrations of the interested molecule. An established waveform for dopamine scans from −0.4 to 1.3 V at a scan rate of 400 V/s. By manipulating the waveform, the detection of cortisol may be performed. The new waveform for cortisol that was used throughout the experiment scanned from −0.5 to −1.2 V at 400 V/s, which will highlight the reduction peak instead of the oxidation peak. The buffer was continually pumped, which washed the tip of the electrode at 1.0 mL per minute. For each trial run, a 0.2 mL volume of cortisol was injected. The electrode was backfilled with 0.1 M KCl solution, which created the connection between the headstage, and equilibrated the electrode by applying the waveform before testing.

### 2.4. Simulated Urine Measurement

Simulated urine was purchased from Carolina Biological, Burlington, NC. We adjusted the pH to 7.0 to make it comparable to the PBS buffer. The simulated urine was diluted in a 7.0 pH PBS buffer using a 1:10 ratio in PBS buffer. The mixture was adjusted to a pH of 7.0.

### 2.5. Statistical Analysis

Statistical analysis was performed with Graphpad PRISM 9 and Excel. Data were exported from HDCV and imported to an Excel file. After being converted into the Excel file, the data were then exported into Graphpad PRISM 9 for further analysis. All of the graphs/figures were created using Graphpad PRISM 9. Some of the data were normalized by dividing all of the data by the greatest value of the particular dataset.

## 3. Results and Discussion

**Scheme 1:**
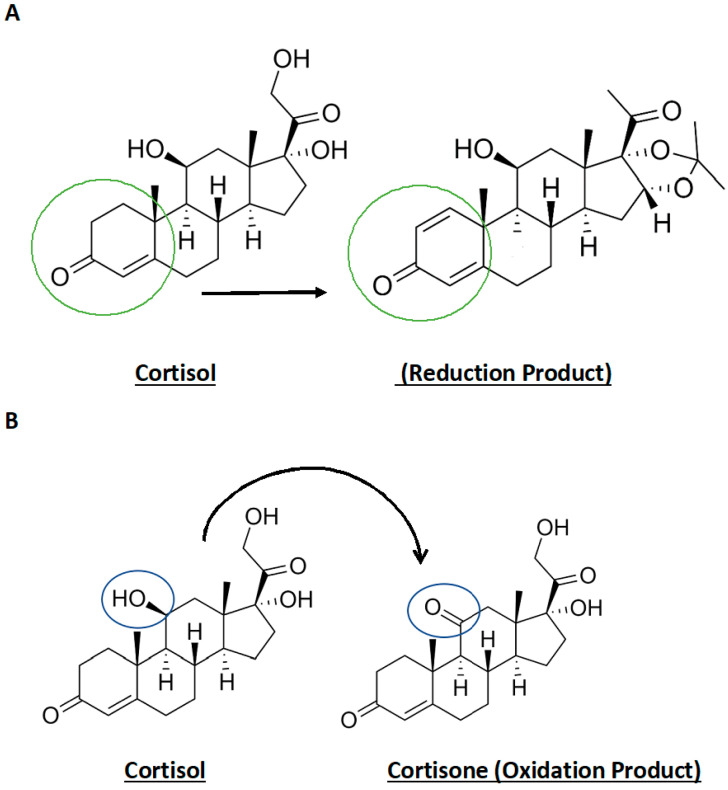
Proposed redox, reduction (**A**) and oxidation (**B**) schemes for cortisol on carbon electrode or CFME surfaces using electrochemical techniques such as FSCV at a physiological pH of 7.0 [12,31].

In order to establish a physiological sensor for cortisol’s redox reactions, we developed several waveforms to either reduce or oxidize cortisol at the electrode surface. However, through waveform method development, we found that cortisol is most likely reduced using carbon electrodes and FSCV [12]. The sensitivity of cortisol was found to be 0.87 ± 0.055 (nA/μM), n = 5. Using FSCV, waveforms scanning toward the positive direction are likely to oxidize the molecule, whereas waveforms scanning toward negative potentials are mostly likely to reduce the molecule. In cortisol’s oxidation reaction, in Scheme 1B, the alcohol group is oxidized to a carbonyl group. In cortisol’s reduction reaction, in Scheme 1A, the fused six-membered ring with a carbonyl group is reduced by the addition of a pi bond. The waveform for cortisol detection scans toward negative potentials and reduces cortisol according to Scheme 1A where electron transfer is more facile for reduction at the carbon fiber electrode surface [31].

**Figure 1:**
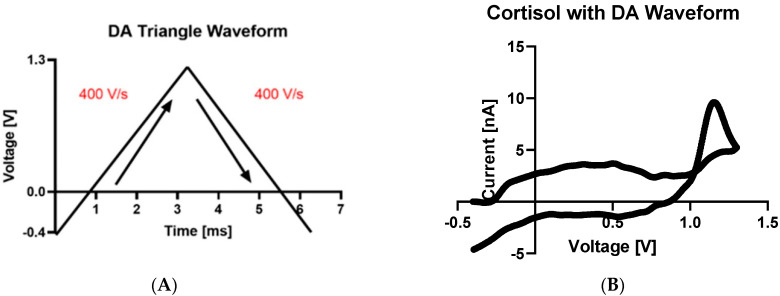

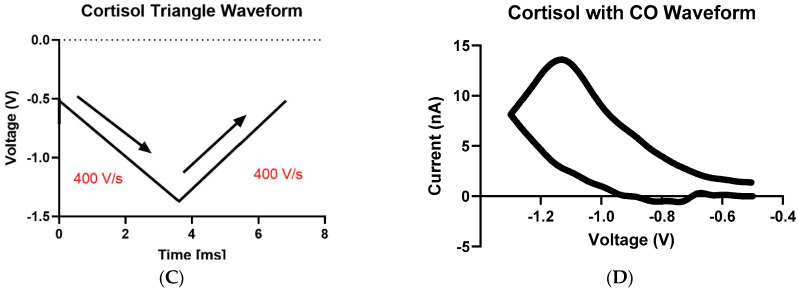
Waveform development to optimize the detection of cortisol. Cortisol detection was compared between the dopamine (DA) waveform and cortisol (CO) waveform. (**A**) Dopamine triangle waveform with (**B**) cortisol CV using dopamine waveform. (**C**) Inverted cortisol triangle waveform with (**D**) cortisol example CV using developed cortisol waveform.

We directly compared the new developed waveform with the established Dopamine (DA) triangle waveform (scanning from −0.4 to 1.3 V at 400 V/s) to determine the most optimal method to measure cortisol (Figure 1A) [32]. The waveform that scanned in the positive direction did not enable cortisol to be detected efficiently and consistently, therefore eliminating the oxidation hypothesis. Prior studies proposed using a waveform that scans from −0.5 to −1.2 V at 400 V/s to detect cortisol with square wave voltammetry [12]. As shown in example CVs (Figure 1B), cortisol detection was not possible when scanning toward the positive direction where no strong oxidation peak was observed. However, utilizing the cortisol waveform (Figure 1C) that scanned from −0.5 to −1.2 V, we observed clear redox activity and a reduction peak at approximately −1.0 V (Figure 1D). Therefore, we hypothesize that the reduction of cortisol is more facile at the electrode surface utilizing FSCV than oxidation is, even though mechanisms exist for both the reduction and oxidation of cortisol, which is redox active at carbon electrode surfaces.

**Figure 2:**
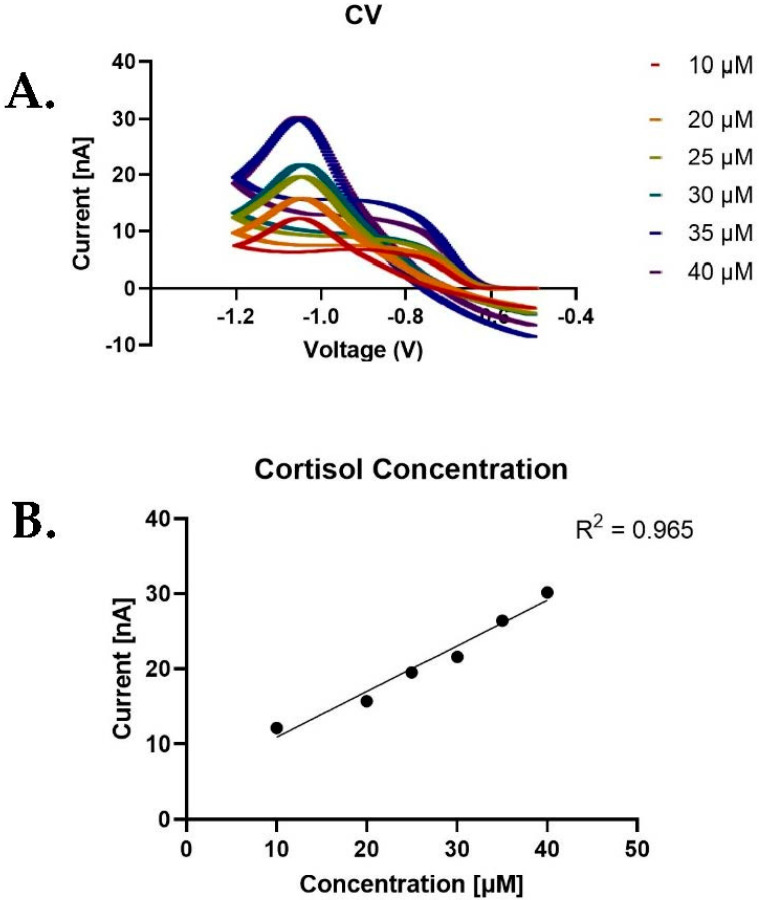
Example of cortisol cyclic voltammograms (CVs) and current concentration dependence using the triangle waveform with increasing concentrations. (**A**) Example of cortisol CVs with increasing concentrations of 10–40 μM. (**B**) Graphs depicting the linear relationship of cortisol concentration vs. current (n = 4).

We optimized the detection of cortisol using the triangle waveform that scanned toward the negative potentials from −0.5 to −1.2 V at 400 V/s (Figure 2A). We proposed that scanning toward negative potentials allows for the electro-reduction to occur at the electrode surface and for cortisol to be detected at lower limits of detection. Cortisol detection was performed at concentrations ranging from 10 to 40 μM using a serial dilution in a solution of PBS buffer pH at 7.0. We hypothesize that unlike dopamine’s sensitive detection at CFMEs, cortisol was only sensitive at higher concentrations due to challenges in electron transfer kinetics and adsorption at the CFME surface.

Measurements below 10 μM were not clearly measured, while concentrations above 40 μM also showed inconsistent data and currents were not linearly increasing, which was possibly due to saturation at the electrode surface yielding an asymptotic relationship on the concentration vs. current curve. Between 10 and 40 μM, current and concentration showed a linear relationship (Figure 2B) similar to other molecules measured with FSCV, which was possibly due to cortisol being adsorption-controlled on the electrode surface [33,34]. At concentrations of 50 μM and higher, cortisol becomes saturated at the surface of the electrode, which produces an asymptotic curve and non-linear currents. Concentration experiments show that cortisol concentration has a linear relationship with respect to the peak current up to 40 μM. Moreover, cortisol CV current was observed to have a steady response on the surface of the electrode over time without much current fluctuation.

**Figure 3:**
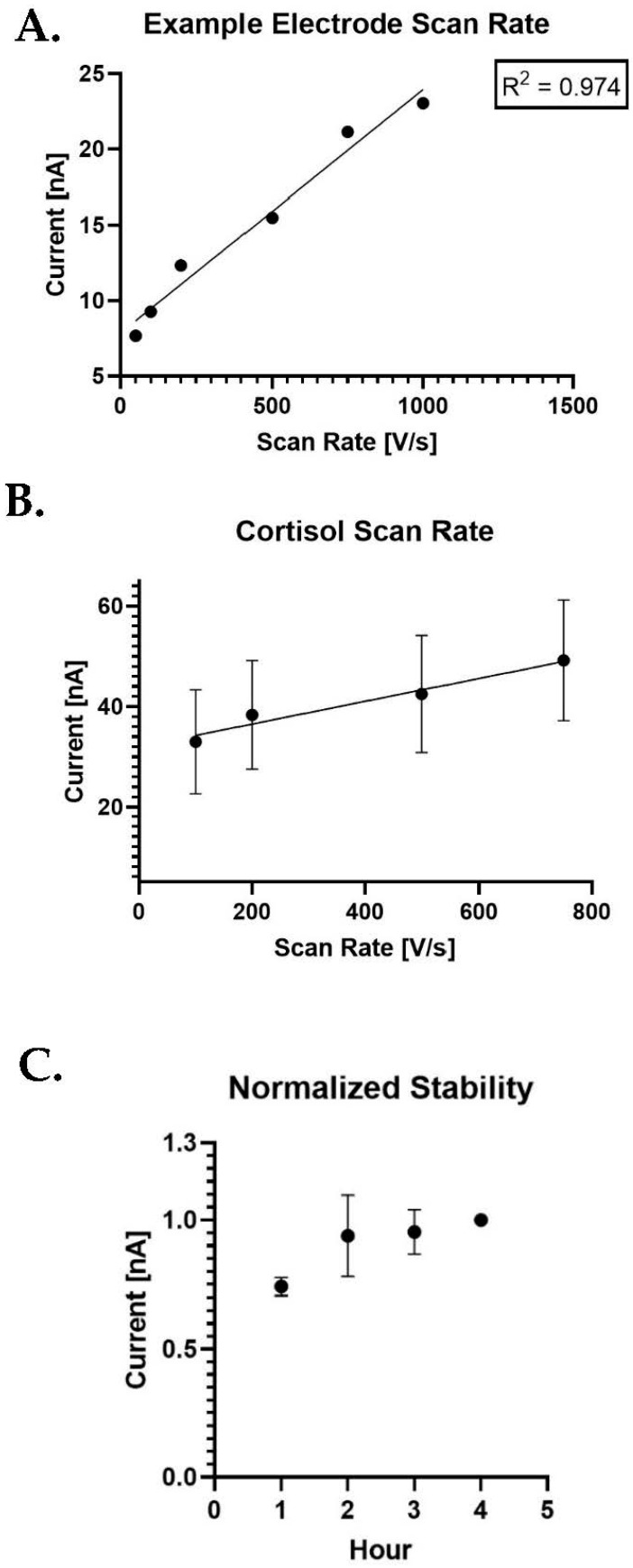
Adsorption control experiments of cortisol on CFMEs. (**A**) Example of electrode scan rate experiment plotting peak current vs. scan rate. (**B**) Cortisol scan rate experiment (n = 4) plotting peak reductive current vs. scan rate 100 to 750 V/S. (**C**) Cortisol stability peak current detection over time (n = 3) with normalized stability over 4 h at a constant concentration of 10 μM.

For a typical experiment, we measure cortisol at a scan rate of 400 V/s. However, we wanted to test the effect of scan rate on the peak current to determine whether cortisol was adsorption or diffusion-controlled to the electrode surface. We began testing by increasing the scan rates from 100 to 750 V/s. As shown in Figure 3C, we observed a highly linear dependence between scan rate and current ($R^{2}$ = 0.970), which denotes a strong correlation between an increase in the scan rate and current of cortisol. This is comparable to the measurement of other biomolecules such as dopamine and serotonin that have a high linear dependence between scan rate and peak current, thus denoting adsorption control of cortisol at the carbon fiber surface [35].

The stability of cortisol was tested over four hours to determine how steady the current response would be after repeated injections in the flow cell, which is the typical duration of an *in vivo* or *ex vivo* experiment. As depicted in Figure 3C, cortisol was measured at a constant concentration of 10 μM and injected every four hours. A stable peak current response was observed throughout the duration of the experiment, which showed that the measurement of cortisol was reliable and reproducible over an extended period of time. After one hour of applying the waveform, the sensitivity of the electrode was enhanced and overoxidized as the waveform etched the electrode surface by breaking carbon–carbon bonds, increasing surface roughness, and functionalizing the electrode with negatively charged surface oxide groups [36,37]. This enhanced the sensitivity toward cortisol adsorption while increasing the surface roughness of the electrode surface.

**Figure 4:**
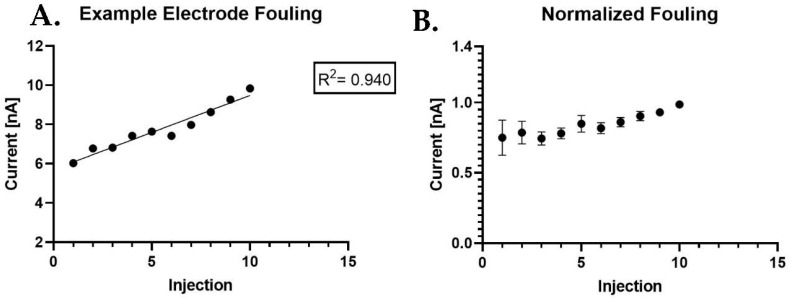
Cortisol fouling experiments. Repeated injections of cortisol display relative increases in current upon repeated (n = 3). Cortisol injections over the course of 300 s (5 min). Injections of 10 μM cortisol were prepared and used. Cortisol did not appear to foul the electrode surface and the current did not decrease over time. (**A**) Fouling data from an example electrode. (**B**) Fouling data from three signal averaged electrodes with normalized current showing the standard error of the mean (SEM).

Furthermore, fouling experiments were performed over a short period of time, at approximately 300 s (5 min), for a total of ten injections with one injection every30 s. As depicted in Figure 4, the cortisol fouling experiments show relative increases in current upon repeated injections of 10 μM cortisol and not a decrease. In Figure 4, we observed an example electrode having a near linear response ($R^{2}$ = 0.940), which depicts a strong positive correlation between the injection times and the current being relatively stable over 300 s. We hypothesize that the current increases due to the waveform etching the electrode surface, hence increasing the number of adsorption sites and sensitivity over time. The fouling of cortisol is not observed as it is with other molecules such as serotonin [38] or 5-hydroxyindoleacetic acid [39] that have been known to polymerize and bio-foul the electrode surface.

Cortisol’s unique reduction reaction toward negative potentials created challenges for the co-detection with other molecules that oxidize or are optimally measured at positive potentials. Molecules such as serotonin, melatonin, epinephrine, norepinephrine, homovanillic acid, 3-methoxytyramine, and adenosine also oxidize at positive potentials [40]. While each molecule was measured with the original cortisol waveform, a new waveform modification had to be developed to encompass both oxidation and reduction peaks of dopamine and cortisol, respectively. The waveform utilized for cortisol’s negative reduction potential did not enable several molecules to be detected such as monoamine neurotransmitters that are primarily oxidized at positive potentials. With cortisol’s primary reduction peak occurring at approximately −1.0 V, the modified waveform needed to encompass a wider potential range from −1.0 to 1.0 V. Other molecules are oxidized at higher positive potentials or have overlapping peaks with cortisol. In this case, dopamine was used as a test compound due to its common oxidation peak at approximately +0.6 V when scanning at 400 V/s from −0.4 to 1.3 V. The waveform potential window was extended from the range of −0.5 to −1.2 V to the range of −1.2 to +1.2 V to allow for both the oxidation and reduction peaks to appear and not overlap with another. The waveform expansion allowed for the co-detection experiments to be performed by identifying and distinguishing both peaks clearly from one another by their designated peak potentials [41].

**Figure 5:**
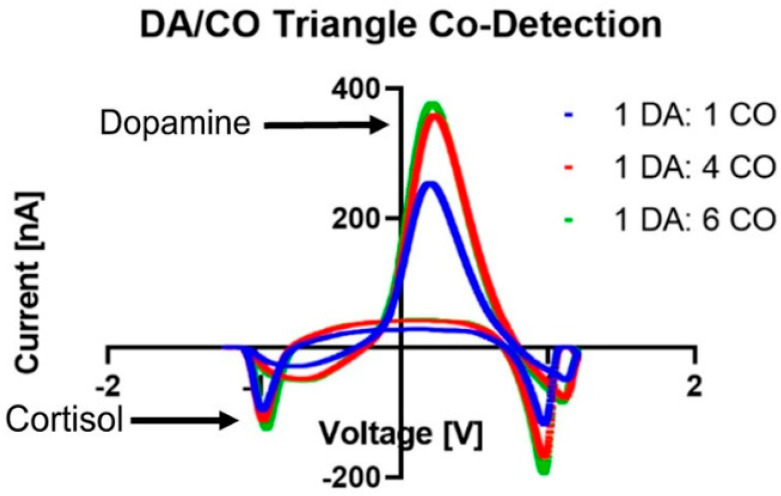
Co-detection experiment of dopamine (DA) and cortisol (CO). The waveform was modified and expanded to visibly identify both peaks from −1.2 to 1.2 V. Cortisol’s reduction peak is observed at −1 V, while dopamine’s oxidation peak is observed at approximately 1 V. Several ratios were used (1:1, 1:4, 1:6) with cortisol concentration increasing and the dopamine concentration held constant.

To properly demonstrate cortisol’s current increase with respect to concentration increase while concentrations of dopamine are held constant, several concentration ratios were developed. Because of dopamine’s relatively high sensitivity to the electrode’s surface, relatively lower concentrations of dopamine were utilized with respect to cortisol to prevent dopamine peak CVs from overshadowing cortisol CVs. Cortisol, however, is less sensitive at the electrode surface with respect to dopamine and requires higher concentration for detection at CFMEs. Dopamine concentration was held constant at 5 μM while cortisol concentrations increased. Ratios of dopamine to cortisol were set at 1:1, 1:4 and 1:6, respectively. By adjusting the concentration ratios, both dopamine and cortisol were co-detected using the extended waveform potential window, observing cortisol’s reduction peak (−1.0 V) increasing in current and dopamine’s oxidation peak (0.6 V) remaining relatively constant (Figure 5). Any changes in dopamine current were hypothesized to be related to a synergistic effect of the contribution of any potential cortisol oxidation to the peak oxidative current. Therefore, we hypothesize that the method allows for the co-detection of cortisol with other important monoamine neurotransmitters such as dopamine that could be differentiated from one another possibly in measurements in complex mixtures such as with *in vivo* or *ex vivo* detection experiments.

**Figure 6:**
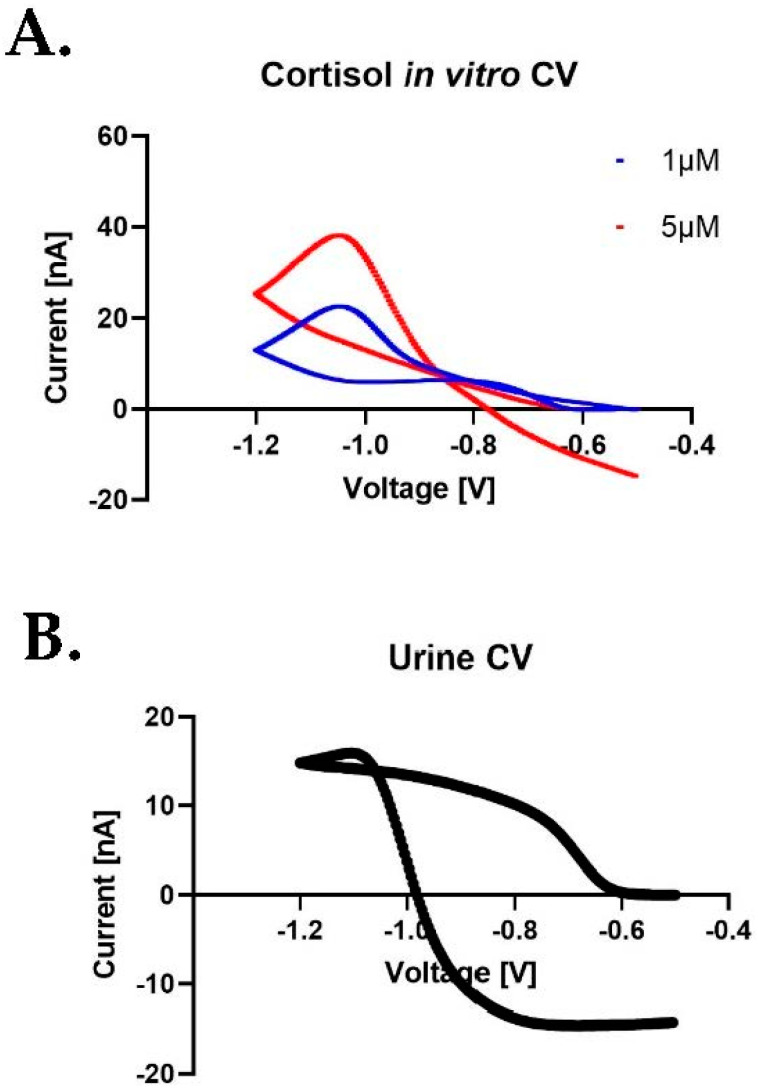
(**A**) Cortisol was injected in simulated urine to provide a proof-of-concept measurement in biological fluid (n = 4). Cortisol was measured at injections 1 μM and 5 μM concentrations, respectively. (**B**) Simulated urine CV absent of cortisol.

We also performed a proof-of-concept experiment to exogenously apply cortisol onto biological samples such as urine. During fight or flight situations, the body releases cortisol, which could potentially be found in sweat, saliva, or urine. Measurements of cortisol in urine are observed in prior studies and are a standard diagnostic for cortisol detection [42] and that of other molecules [43]. Urine was a convenient sample to measure as a biological sample because of its ease of accessibility and due to the ability to alter pH whose fluctuations can be measured at the electrode surface [44]. Urine contains uric acid, urea, and other components that make it slightly more acidic than buffer at a physiological pH. The urine used had a pH of 4, which was diluted with PBS buffer at a 1:10 ratio. Cortisol was exogenously applied at different concentrations (1 μM and 5 μM) in the urine to determine whether uric acid, urea, or other components of the urine hindered the detection of cortisol with CFMEs and FSCV (Figure 6A). We performed the measurements of cortisol diluted in urine to determine whether other electroactive analytes in the urine such as uric acid or other molecules were interfering or deterring cortisol detection. The CV of solely dilute urine was obtained (Figure 6B) and did not yield any definitive peak shape that denotes faradaic current resulting from redox activity electron transfer to the electrode surface.

Cortisol was subsequently measured both in buffer and urine. Tests were conducted with various cortisol concentrations exogenously applied in diluted urine. Samples of 1–5 μM were tested and showed relatively lower currents of cortisol with respect to measurements of cortisol diluted in only buffer. We hypothesize that other molecules within the urine could potentially be interfering with cortisol detection in the urine, hence the lower response. However, cortisol was still measured in the diluted urine, which shows the potential of this technique to be used for measuring cortisol in real patient samples.

The percent recovery was found to be approximately 98% upon increasing the concentration to 5 μM, which shows that the urine does not significantly hinder cortisol detection at higher concentrations (Table 1). We hypothesize that analytes within urine such as urea and uric acid could potentially be interacting with the electrode surface, thus altering the concentration of cortisol measured. Previous studies have shown cortisol to be measured at relatively low concentrations in saliva and urine via capacitive determination [34]. Measurements of real urine samples could potentially contain endogenous levels of cortisol that could be measured with CFMEs and FSCV, which would greatly enhance the utility and usage of this technique.

## 4. Conclusions

In conclusion, the development of methods for the detection of cortisol using carbon electrodes and FSCV has enabled the sensitive and selection detection of cortisol with high spatiotemporal resolution and biocompatibility. Using a triangle waveform, we were able to electro-reduce cortisol on the electrode surface. Cortisol showed strong adsorption control on the surface of the electrode and was differentiated from monoamine neurotransmitters such as dopamine based on the shape and positions of the respective voltammograms. Proof of principle measurements were performed in exogenously applied injections biological fluids such as urine for clinical and translational relevance. Enhancing the detection and measurement of cortisol will help further understand its interaction with the electrode surface and physiological importance *in vivo*. Future studies include determining cortisol’s physiological importance through its measurement in biological fluids during periods of anxiety and possible pharmaceutical treatments.

## Figures and Tables

**Table 1:** Percent recovery of cortisol when measured in urine.

Cortisol (μM) in Dilution	Percent Recovery of Cortisol in Urine
1 μM	53.1%
5 μM	98.5%

## Data Availability

Data available upon request.
